# Efficient Removal of Congo Red, Methylene Blue and Pb(II) by Hydrochar–MgAlLDH Nanocomposite: Synthesis, Performance and Mechanism

**DOI:** 10.3390/nano13071145

**Published:** 2023-03-23

**Authors:** Yang Huang, Wei Yin, Tian-Lei Zhao, Meng Liu, Qi-Zhi Yao, Gen-Tao Zhou

**Affiliations:** 1 CAS Key Laboratory of Crust-Mantle Materials and Environments, School of Earth and Space Sciences, University of Science and Technology of China, Hefei 230026, China; esthy@mail.ustc.edu.cn (Y.H.);; 2 School of Environmental Engineering and Resources, University of Science and Technology of Southwest, Mianyang 621010, China; 3 School of Chemistry and Materials Science, University of Science and Technology of China, Hefei 230026, China; 4 CAS Center for Excellence in Comparative Planetology, University of Science and Technology of China, Hefei 230026, China

**Keywords:** LDH, hydrochar, organic dyes, Pb(II), simultaneous removal

## Abstract

Organic dyes and heavy metals often coexist in industrial effluents, and their simultaneous removal is a grand challenge. Herein, a hydrochar and MgAl layered double hydroxide (HC–MgAlLDH) nanocomposite was prepared via a facile one-step hydrothermal route, and applied to remove anionic Congo red (CR), cationic Methylene blue (MB) and Pb(II) from aqueous solutions. The nanocomposite was formed by interweaving amorphous HC and crystalline MgAlLDH nanoplates and possessed more functional groups, lower zeta potential and larger specific surface area than uncomposited MgAlLDH. Batch removal experiments showed that the components HC and LDH dominated the CR and MB removals, respectively, whereas Pb(II) removal was conjointly controlled by the two components. The maximum Langmuir removal capacities of the nanocomposite to sole CR, MB, or Pb(II) were 348.78, 256.54 or 33.55 mg/g. In binary and ternary systems, the removal capacities of CR and MB only slightly decreased, while the capacity of Pb(II) increased by 41.13–88.61%. The increase was related to the coordination of Pb(II) with the sulfur-containing groups in dyes and the precipitation of PbSO_4_. Therefore, the simultaneous removal of CR, MB and Pb(II) was involved in a synergistic effect, including electrostatic adsorption, π–π interaction, coordination and precipitation. The present work shows that the HC–MgAlLDH nanocomposite has great potential for wastewater integrative treatment.

## 1. Introduction

Organic dyes are widely used in the paper, paint, plastic and textile industries, and usually have complex and various molecular structures, like azo, anthraquinonoid, and heterocyclic groups [1,2]. Due to their widespread use, refractory structures against degradation and high biological toxicity, organic dyes have been regarded as one of the main hazards in industrial wastewater [2,3]. At present, a challenge for the wastewater treatment is that different types of pollutants usually coexist together, significantly increasing the treatment difficulty and cost [4,5]. Anionic Congo red (CR) and cationic Methylene blue (MB) are two typical dyes used in industry and concomitant in the effluents as a central discharge of dye wastewaters [6]. Moreover, lead-containing compounds are often used as mordants, colorants and inorganic pigments in the printing and dyeing industry [7], inevitably leading to the coexistence of Pb(II), CR and MB. However, most technologies are inefficient for simultaneous removal of the multiple pollutants because of their different physicochemical properties (e.g., molecular size and chemical structure) [8,9]. In addition, both the heavy metals and dyes show strong toxicity, environmental persistence and bioaccumulation [10,11]. The environmental persistence makes them accumulate in the environment and consequently contaminate the food chains, and the bioaccumulation in biota causes a health threat to their consumers including humans [10,11]. More seriously, increasing data indicates that the coexisting heavy metal ions and dyes generally pose greater combined toxicity to living organisms than individually [12,13,14]. Hence, it is significant to develop effective approaches for the removal of the coexisting pollutants.

Adsorption is widely adopted to remove organic dyes or heavy metal ions due to its simple process and low cost [15]. Among the adsorbents, layered double hydroxides (LDHs) have recently attracted extensive attention because of their tunable structure, high surface area, facile synthesis, environmental friendliness and low cost. Structurally, LDHs, with a formula of [M^2+^_1−x_M^3+^_x_ (OH)_2_]^x+^(A^n−^)_x/n_·mH_2_O, are stacked by positively charged (M^2+^, M^3+^)(OH)_6_ octahedral layers, interlayer anions (A^n−^), and interlayer water molecules, where M^2+^ and M^3+^ are divalent (e.g., Mg^2+^, Co^2+^, Cu^2+^, Zn^2+^ and Ni^2+^) and trivalent (e.g., Al^3+^, Cr^3+^ and Fe^3+^) metal cations, x represents the ratio of M^3+^/(M^2+^ + M^3+^) and is usually between 0.18 and 0.33, and A^n−^ includes CO_3_^2−^, SO_4_^2−^ and NO_3_^−^ [16,17]. The structural features endow LDHs with a high isoelectric point and strong interlayer anion exchangeability [18,19], which could make them become an excellent adsorbent for the removal of anionic CR. However, the adsorption of LDHs to cationic MB and Pb(II) is greatly limited due to electrostatic repulsion [20]. Modification of LDHs with other materials may be a feasible method to construct multifunctional active sites for various types of pollutants.

Hydrochar (HC), as an incomplete carbonized material, can be easily derived from cheap and widespread saccharides or other biomass at lower temperatures relative to other carbonaceous materials [21,22,23]. More importantly, HC exhibits a high adsorption affinity for heavy metal cations and cationic dyes in solution due to its abundant oxygen-bearing functional groups and high negative charge nature [24,25,26,27,28]. Therefore, the successful composition of LDH and HC may donate the material with better performance for simultaneous removal of the multiple pollutants. In particular, several recent studies on the synthesis of HC-modified LDH composites and their removals to cationic and anionic pollutants further supported such potential. For example, Memon et al. synthesized an HC and CoAlZnLDH composite through the coprecipitation of Co^2+^, Al^3+^ and Zn^2+^ in the presence of as-prepared HC at 60 °C, and found that the composite can simultaneously adsorb cationic and anionic dyes [29]. Dat et al. prepared MgAlLDH-capped HC spheres by hydrothermal treatment of pre-synthesized HC and MgAlLDH mixture for removal of single cationic or anionic dye [30]. Zhang et al. fabricated HC-coated MgAlLDH composites by a two-step hydrothermal method to remove either heavy metals or anionic dyes [31]. Despite all this, the removal behavior in the multi-pollutant system of dye and heavy metal was not involved, and the syntheses underwent a two-step process [30,31]. Therefore, it is necessary to develop a more simple and more economical synthesis of HC and LDH composite materials and to explore the removal performance and mechanism in multi-pollutant systems.

Herein, a series of HC–MgAlLDH nanocomposites were prepared by a one-step hydrothermal route and used for the adsorptive removal of CR, MB and Pb(II). The structural characteristics of the nanocomposites were comprehensively characterized, and the removal abilities of the three targets were systematically evaluated by batch adsorption experiments. The removal mechanisms were further explored based on the results of batch experiments and the analyses of post-adsorbents. Overall, the good removal performance of the HC–MgAlLDH nanocomposite showed potential for practical applications.

## 2. Materials and Methods

### 2.1. Materials

Magnesium nitrate hexahydrate (Mg(NO_3_)_2_·6H_2_O), aluminum nitrate nonahydrate (Al(NO_3_)_3_·9H_2_O), sodium hydroxide (NaOH), sodium nitrate (NaNO_3_), sodium carbonate (Na_2_CO_3_), glucose (C_6_H_12_O_6_), nitric acid (HNO_3_), Congo Red (C_32_H_22_N_6_Na_2_O_6_S_2_), Methylene Blue (C_16_H_18_ClN_3_S·3H_2_O) and lead nitrate (Pb(NO_3_)_2_) were purchased from Sinopharm Chemical Reagent Co., and are of analytical grade. Deionized water was used in all experiments.

### 2.2. Preparation of HC–MgAlLDH Nanocomposites

Hydrothermal synthesis has been confirmed as a promising method for HC and LDH, respectively [32,33]. In the present work, HC–MgAlLDH nanocomposites (HC–MgAlLDH) were prepared by the one-pot hydrothermal synthesis. In a typical procedure, 0.004 mol of Mg(NO_3_)_2_·6H_2_O and 0.002 mol of Al(NO_3_)_3_·9H_2_O were dissolved in 50 mL of deionized water to obtain solution A, with a Mg^2+^/Al^3+^ molar ratio of 2. Solution B was prepared by dissolving 0.5 mol of NaOH and 0.05 mol of Na_2_CO_3_ in 250 mL of deionized water. Solution A was added dropwise into a beaker containing 10 mL of 100 g/L glucose (1.0 g) solution at room temperature and vigorous magnetic stirring, and the pH was adjusted to 9.5–10.0 using ca. 10 mL of solution B. Subsequently, the obtained suspension was transferred into a 100 mL of Teflon-lined stainless-steel autoclave. The autoclave with the suspension was sealed and placed into a programmed furnace to be kept at 180 °C for 48 h. After that, the autoclave was allowed to cool naturally to room temperature, and the resultant product was separated by centrifugation, rinsed with deionized water several times, and dried in a vacuum oven at 60 °C for 24 h. For comparisons, HC and MgAlLDH were synthesized under the same conditions, but without the addition of LDH-forming ions or glucose, respectively. In addition, the concentration of glucose was adjusted from 50 to 200 g/L to obtain the nanocomposites with different contents of HC, and the samples are denoted as xHC–MgAlLDH, where x represents the initially added weight of glucose, i.e., 0.5, 1.0, 1.5 and 2.0 g.

### 2.3. Characterizations

The synthesized products were identified by powder X-ray diffraction (PXRD) using a Rigaku diffractometer (40 kV, 30 mA) with Cu Kα radiation (λ = 0.154056 nm). Morphology and structure analyses were performed on a Zeiss Ultra 55 scanning electron microscope (SEM) and Libra 200FE transmission electron microscope (TEM) with an accelerating voltage of 5 and 200 kV, respectively. Analyses of energy dispersive spectroscopy (EDS) were performed on the SEM. X-ray photoelectron spectra (XPS) were obtained on a Thermo ESCALAB 250 XPS spectrometer with Al Kα radiation. N_2_ adsorption–desorption isotherms were determined by Quantachrome, Autosorb-1MP analyzer at the liquid-nitrogen temperature of 77 K. Specific surface areas were acquired according to the multipoint Brunauer–Emmett–Teller (BET) model, and total pore volumes and pore sizes were calculated based on the Barret–Joyner–Halenda (BJH) model. Zeta potential was determined by micro-electrophoresis using a Malvern Zetasizer Nano Zs90 zeta potential analyzer. The suspensions for zeta potential analyses contained 0.5% sample and 0.01 mol/L NaNO_3_, and the pH was adjusted using 0.1 mol/L NaOH and 0.1 mol/L HNO_3_. Fourier transform infrared (FT-IR) spectra were recorded on a PerkinElmer Fourier transform infrared spectroscopy, in the scan range of 4000–400 cm^−1^.

### 2.4. Removal Experiments

Batch removal experiments were performed at 25 °C to evaluate the effects of initial solution pH, contact time, and initial CR, MB, or Pb(II) concentration on the removal by the synthesized adsorbents. Briefly, 10 mg of the adsorbent was added into an Erlenmeyer flask containing 20 mL of the working solution, and the adsorption was performed in an orbital shaker (TS2102-C) at 150 rpm. After a pre-set time, the suspension was centrifuged by high-speed centrifugation at 8000 rpm for 5 min. The concentrations of CR and MB in the supernatant were measured with a Thermo Fisher evolution 300 UV-Vis spectrophotometer at the maximum absorption wavelengths of 494 and 662 nm, respectively. The residual concentration of Pb(II) was determined by ICP-OES (PerkinElmer, optima 8300). The removal capacity *q*_t_ (mg/g) for each sorbate was calculated by the following equation:

(1) $$q_{t}=\frac{\left(C_{0}-C_{t}\right)\times V}{m}$$

where *C*_0_ is the concentrations of CR, MB, or Pb(II) at the initial time (mg/L), *C_t_* is the concentration at any time *t*, *V* is the volume of solution (L), and *m* is the mass of adsorbent (g).

## 3. Results

### 3.1. Characterization of HC–MgAlLDH Nanocomposites

#### 3.1.1. Phase Composition and Crystallinity

**Figure 1:**
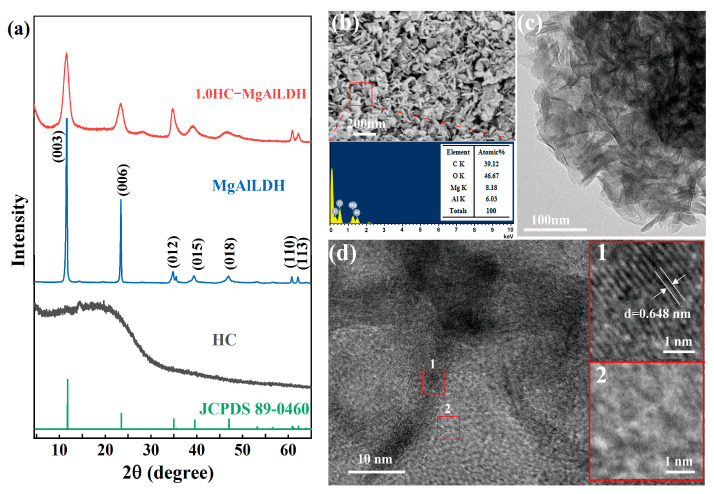
XRD patterns (**a**) of the synthesized HC, MgAlLDH and HC–MgAlLDH; SEM (**b)**, TEM (**c**) and HRTEM (**d**) images of the HC–MgAlLDH, with the corresponding EDS spectrum in panel b.

To examine the phase composition and crystallinity of the synthesized samples, XRD analyses were first performed. Figure 1a depicts the representative XRD patterns of the samples. The sample without LDH-forming ions shows a broad hump with low intensity at 2θ 15–25°, indicating that the synthesized product is amorphous carbon [34,35]. Therefore, the HC is successfully synthesized. The sample only with LDH-forming ions exhibits seven diffraction peaks at 2θ 11.6, 23.5, 34.8, 39.4, 46.8, 60.8 and 62.1°, which can be indexed to the (003), (006), (012), (015), (018), (110) and (113) diffractions of trigonal hydrotalcite (Mg_0.667_Al_0.333_(OH)_2_(CO_3_)_0.167_)·0.5H_2_O with lattice parameters a = 3.046 Å, c = 22.772 Å, and space group *R*3m (JCPDS file 89-0460). This demonstrates that the MgAlLDH was hydrothermally formed under the current conditions. For sample HC–MgAlLDH, all characteristic diffractions of the LDH are reliably detected, but the diffractions become much broader relative to the MgAlLDH, indicating that the LDH in the composite has lower crystallinity and smaller crystallite size. Further estimations of the crystallite sizes by the Scherrer equation show that the average size of the LDH crystallites in the composite is 61 nm, smaller than the MgAlLDH (311 nm) formed in the absence of glucose, indicating that the glucose and/or its derivatives limit crystal growth of the LDH.

#### 3.1.2. Morphology and Microstructure

The morphology, microstructure and size of the prepared materials were determined by SEM and TEM observations. As can be seen from the SEM (Figure 1b) and TEM (Figure 1c) images, the HC–MgAlLDH is composed of numerous interweaved nanoplate-like structures with sub-100 nm sizes. Under the high-resolution bright-field mode of TEM (Figure 1d), the interweaved structures clearly show two distinct contrasts, the dark regions with obvious lattice fringes (e.g., box 1), but the light regions with an amorphous nature (e.g., box 2). Furthermore, the lattice fringes correspond well to the lattice plane of MgAlLDH, e.g., the 0.648 nm lattice fringe assigned as the (003) plane of trigonal hydrotalcite (box 1 in Figure 1d), whereas the amorphous materials can be assigned to the HC by combining the XRD results (e.g., Figure 1a). The high-resolution TEM images also show that the LDH and HC tightly coalesce together, indicating the successful formation of the HC–MgAlLDH nanocomposite. By contrast, the MgAlLDH synthesized without the addition of glucose presents a characteristic hexagonal sheet-like shape of LDH (e.g., Figure S1a) and a larger size (diameters of 200–1000 nm and a thickness of ~50 nm) than the LDH in the nanocomposite. This reveals that the growth of LDH crystals was inhibited with the addition of glucose, supporting the XRD analyses. Similarly, the HC synthesized without the LDH-forming ions exhibits a different morphology from that in the nanocomposite. The HC is nanospheres with smooth surfaces and uniform diameters of 300 nm (Figure S1b), showing the characteristics of the hydrothermal carbonization product of glucose [36]. In addition, the EDS results confirm that the nanocomposite (39.12%) has a much higher content of C than the LDH (15.48%) (Figure 1b and Figure S1a), further confirming the composition of HC and LDH. The HC also contains 19.04% O (Figure S1b), indicating that the nanocomposite has considerable amounts of O-rich functional groups.

#### 3.1.3. Surface Chemistry and Zeta Potential

**Figure 2:**
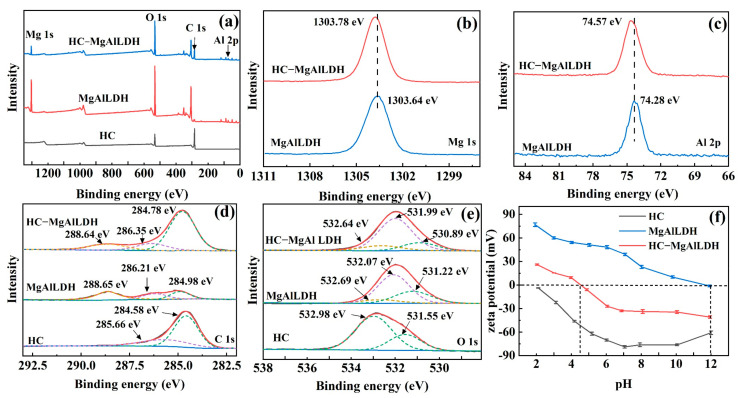
XPS spectra and zeta potentials of the synthesized HC, MgAlLDH and/or HC–MgAlLDH: XPS survey spectra (**a**); high-resolution Mg 1s (**b**), Al 2p (**c**), C 1s (**d**) and O 1s (**e**) XPS spectra; zeta potentials (**f**).

The surface chemical states and elemental composition of the samples were identified by XPS analysis. The survey spectra reveal that O, C, Mg and Al elements were detected in the prepared MgAlLDH and nanocomposite (Figure 2a). However, compared with the LDH, the nanocomposite shows a stronger C 1s peak (Figure 2a), indicating that a large number of carbon-containing materials were formed. In addition, the high-resolution scans of Mg 1s (Figure 2b) and Al 2p (Figure 2c) regions show that their electron binding energies shifted from 1303.64 and 74.28 to 1303.78 and 74.57 eV after the HC composition, respectively, confirming that the HC was chemically bound to the MgAlLDH. Moreover, the high-resolution C 1s spectrum of the nanocomposite can be deconvoluted into three regions centered at 284.78, 286.35 and 288.64 eV (Figure 2d), corresponding to CH_x_/C–C/C=C, C–O and O–C=O, respectively [37]. The CH_x_/C–C/C=C and C–O components have larger peak areas than the O–C=O, indicating that more C–O and CH_X_/C–C/C=C functional groups formed on the nanocomposite surface. The three O 1s subpeaks at approximately 530.89, 531.99 and 532.64 eV (Figure 2e) can be assigned to O=C, Mg–O/Al–O and –OC/COOR, respectively [34]. Overall, the XPS results confirm that the HC and MgAlLDH are composited by chemical interactions, and the nanocomposite possesses abundant O=C and –OC/COOR groups.

Their surface properties were further examined by a zeta potential analyzer. As shown in Figure 2f, the zeta potential of MgAlLDH is always positive at pH 2–12, probably due to the permanent positive charge of the primary lamellar plate from the isomorphic replacement of Mg^2+^ by Al^3+^ in LDH layers [38]. By contrast, the nanocomposite shows a much lower zeta potential at all the pH values, indicating that the modification with HC introduced oxygen-bearing functional groups with negative charges (e.g., –COO^−^) to the surface of the nanocomposite. Thus, it can be anticipated that the combination of LDH with HC could improve the adsorption abilities of LDH to cationic MB and Pb(II).

#### 3.1.4. Specific Surface Area and Pore Structure

**Figure 3:**
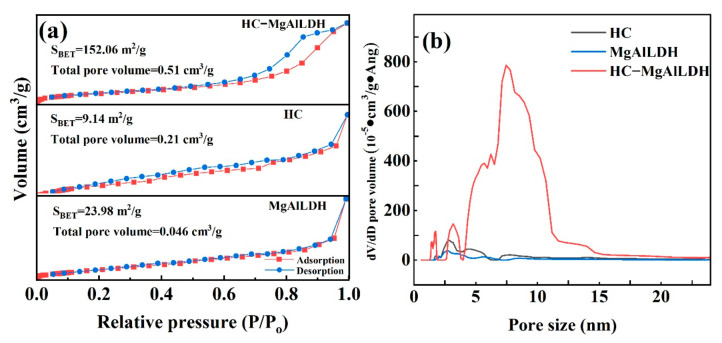
N_2_ adsorption−desorption isotherms (**a**) and pore size distributions (**b**) of the MgAlLDH, HC and HC–MgAlLDH.

The specific surface area and pore size distribution for the samples were determined by N_2_ adsorption–desorption tests. The results are shown in Figure 3. For the HC–MgAlLDH, HC and MgAlLDH, all of the adsorption–desorption curves exhibit type IV isotherms (Figure 3a), revealing the presence of mesopores in the three materials. Nevertheless, the nanocomposite (Figure 3a) presents higher N_2_ uptakes and a more remarkable hysteresis loop of H3 type in the range of 0.45–1.00 *P*/*P*_0_, indicating that the nanocomposite has a larger specific surface area and more slit-shaped pores [39]. The calculated BET-specific surface area (*S*_BET_) of the nanocomposite is 152.06 m^2^/g (Figure 3a), which is sharply higher than the HC 9.14 m^2^/g, and the MgAlLDH 23.98 m^2^/g. The pore volume (*V*_pore_) of the nanocomposite is 0.51 cm^3^/g, much larger than the *V*_pore_ of the MgAlLDH 0.046 cm^3^/g or the HC 0.21 cm^3^/g (Figure 3a). In addition, the pore size distributions also demonstrate the significant porous structure in the nanocomposite, mainly in the range of 4–11 nm (Figure 3b). The increases in *S*_BET_ and *V*_pore_ may be conducive to the adsorption of CR, MB and Pb(II) onto the HC–MgAlLDH nanocomposite.

### 3.2. Removal Performance of CR, MB and Pb(II)

#### 3.2.1. Optimizing the Compositional Ratio of HC and MgAlLDH

**Figure 4:**
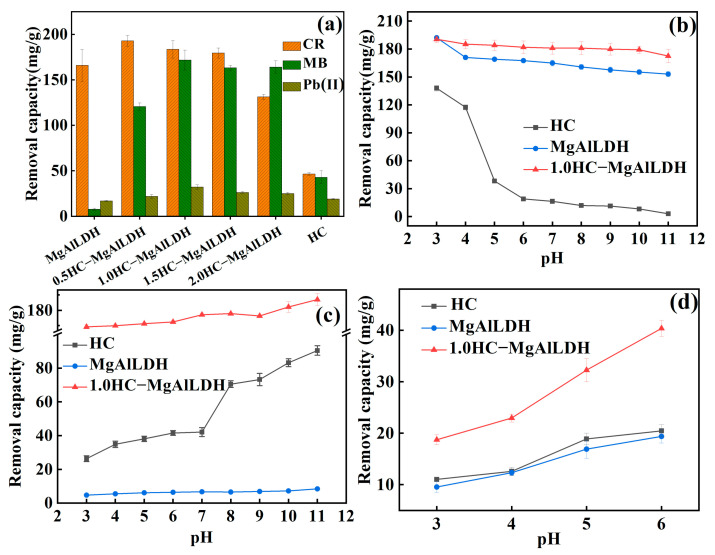
Removal capacities of the HC–MgAlLDH nanocomposites with different HC contents to single CR, MB and Pb(II) (**a**); effect of pH on the removal of single CR (**b**), MB (**c**) and Pb(II) (**d**) by the 1.0HC–MgAlLDH nanocomposite, MgAlLDH nanoplates and HC nanospheres.

In order to screen the optimal nanocomposite, the HC, MgAlLDH, and their nanocomposites with different doping amounts of HC were used to remove single CR, MB and Pb(II) at contact time 12 h, pH 5, initial concentrations 100 mg/L for CR or MB, and 50 mg/L for Pb(II). As shown in Figure 4a, the removal capacities of the adsorbents to the three adsorbates all firstly increase, and then decrease with the HC contents. Nevertheless, it can be also seen from Figure 4a that 1.0HC–MgAlLDH has a relatively high removal capacity either for CR (183.72 mg/g), MB (171.88 mg/g), or Pb(II) (32.26 mg/g), despite the highest CR removal 193.01 mg/g achieved by 0.5HC–MgALDH. Therefore, the adsorbent 1.0HC–MgAlLDH was chosen in the following removal experiments.

#### 3.2.2. Effect of Solution pH

The solution pH can significantly affect the adsorption performance because it may affect the surface charge of adsorbents and the species of sorbates [39]. Generally, Pb(II) can precipitate as Pb(OH)_2_ in an alkaline solution ($K_{\mathrm{sp},\mathrm{Pb}{\left(\mathrm{OH}\right)}_{2}}=10^{-15.00}$) (Figure S2). Thus, for the single pollutant, the initial pH values ranging from 3 to 6 were selected for Pb(II) removal, and a wider pH range of 3−11 for CR and MB, but the other parameters including contact time, and the initial concentrations of CR, MB and Pb(II) were kept. The pH-dependent removal profiles for single CR, MB and Pb(II) by the nanocomposite are depicted in Figure 4b–d, respectively, and compared with the removals by LDH and HC. For the three sorbates, the nanocomposite shows much higher removal capacities than LDH and HC at all the pHs (Figure 4b–d), indicating that the composition of MgAlLDH and HC enhanced the removal performance. Specifically, the removal capacity to CR by the nanocomposite was as high as 172.47–190.49 mg/g at pH 3–11, despite a slight decrease with the increase of pH (Figure 4b). The high removal of CR should be dominated by the component LDH in the nanocomposite because the unmodified LDH also have a similar but slightly inferior removal (Figure 4b). LDH, as a typical material with positively charged host layers, can adsorb anionic pollutants by strong electrostatic attraction [40]. In our study, the LDH always has positive zeta potentials over the pH from 3 to 11 (Figure 2f), and thus can generate strong adsorption to anionic CR. Nevertheless, the zeta potentials of the LDH decrease with increasing pH (Figure 2f). As a result, the decline in CR removal was observed either for the LDH or the nanocomposite in the scenario with higher pHs (Figure 4b). It is worth noting that the higher CR removal by the nanocomposite relative to the LDH (Figure 4b) should be attributed to its higher porosity and surface area (Figure 3). Similarly, for cationic MB, the negatively charged nanocomponent HC should significantly contribute to the removal due to the low removal of the LDH to MB (Figure 4c). The increasing removal capacity from 163.82 to 190.58 mg/g with a pH from 3 to 11 (Figure 4c) should result from the enhancement of electrostatic attraction (Figure 2f). The higher porosity and surface area could also be responsible for the higher removal capacities of the nanocomposite. However, the Pb(II) removal capacities by the nanocomposite appear to be the sum of the capacities by the nanocomponents LDH and HC (Figure 4d), implying that the two nanocomponents conjointly controlled the Pb(II) removal. In addition, the Pb(II) removal increases obviously from 18.72 to 40.36 mg/g with the pH from 3 to 6. This could be attributed to the deprotonation of –COOH to form –COO^−^ and less competitive adsorption between Pb(II) and H^+^ at higher pHs [34]. As a result, the wide and effective pH response could make the HC–MgAlLDH nanocomposite become a versatile adsorbent for cationic and anionic dyes and heavy metals.

#### 3.2.3. Effect of Contact Time

**Figure 5:**
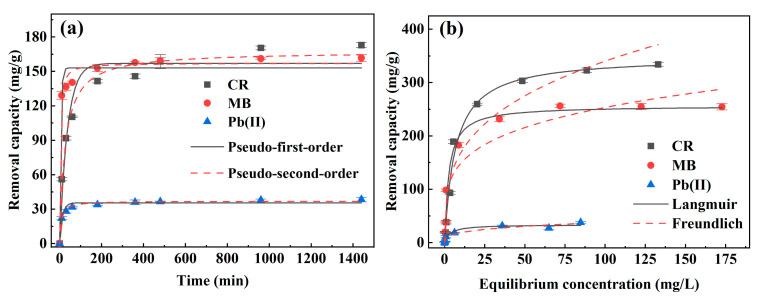
Adsorption kinetics (**a**) and isotherms (**b**) of single CR, MB and Pb(II) by the HC–MgAlLDH nanocomposite.

The effects of contact time on CR, MB, or Pb(II) removal by the nanocomposite were investigated from 10 to 1440 min at initial pH 5, initial concentrations 100 mg/L for CR or MB, and 50 mg/L for Pb(II). The time-course removal profiles were depicted in Figure 5a. For the three targeted species, the removal capacities all increased very rapidly within the first 60 min, indicating that the nanocomposite possessed massive and multifunctional active sites for anionic CR and cationic MB and Pb(II). Subsequently, the increases were slow and adsorption saturations approached after about 360 min, implying that the active sites on the nanocomposite were almost completely occupied. Thus, a contact time of 720 min was used in the other batch experiments for a sufficient equilibrium state. Furthermore, the removal processes were analyzed by the nonlinear pseudo-first-order and pseudo-second-order kinetic models [41]. Detailed information on the models is provided in the Supplementary Material (Text S1), and the applicability was judged by the correlation coefficients (*R*^2^). The results (Table S1) show that the pseudo-second-order model gives rise to greater *R*^2^ values (all exceeding 0.98) than the pseudo-first-order, indicating that the removal of CR, MB, or Pb(II) onto the HC–MgAlLDH nanocomposite follows the pseudo-second-order kinetic model.

#### 3.2.4. Adsorption Isotherm

Figure 5b depicts the adsorption isotherms of CR, MB or Pb(II) determined at pH 5.0, contact time 12 h, initial concentrations 10–300 mg/L for CR or MB and 1–100 mg/L for Pb(II). It can be seen that their removal capacities increased rapidly with the equilibrium concentrations at the initial stage and then approached the maximum value. The fittings to the adsorption data by the Langmuir and Freundlich isothermal models show that all the adsorptions followed well the Langmuir model (Figure 5b and Table S2), revealing a monolayer adoption characteristic of the three sorbates on the HC–MgAlLDH [42]. Furthermore, the theoretical maximum removal capacities (*q_m_*) determined by the Langmuir model for sole CR, MB and Pb(II) are 348.78, 256.54 and 33.55 mg/g, respectively, which are higher than partial previously reported LDH-based adsorbents (Table S3). Specifically, the *q_m_* for CR is higher than those of MgAlLDH (111.11 mg/g) [33], borate intercalated MgAlLDH (166.39 mg/g) [43], ZnFe_2_O_4_/MgAlLDH (294.12 mg/g) [44], and MgAlLDH modified diatom (305.8 mg/g), [45]. The *q_m_* for MB exceeds the values of ZIF-67/CoAlLDH (57.24 mg/g [46]), NiFeLDH decorated montmorillonite (99.18 mg/g) [20], dodecyl sulfate modified ZnAl LDH (113.00 mg/g) [47], and spherical HC capped MgAlLDH (226.00 mg/g) [30]. For Pb(II) removal, the nanocomposite also outperforms the MgAlLDH (16.93 mg/g) [48], MgFeLDH (18.45 mg/g) [19], and tartrate intercalated MgAlLDH (8.40 mg/g) [49]. Therefore, the prepared HC–MgAlLDH nanocomposite exhibited a good removal performance for single CR, MB and Pb(II).

#### 3.2.5. Reusability

The reusability of an adsorbent is important for its practical application in wastewater treatment. To verify the reusability of the HC–MgAlLDH nanocomposite, the post-adsorbents were regenerated by desorption with eluent agents. Specifically, 0.1 M NaOH, ethanol, and 0.01 M HCl were used to desorb the adsorbed CR, MB, and Pb(II), respectively [50,51,52]. The regenerated HC–MgAlLDH nanocomposites were then reused for four cycles. The results are presented in Figure S3. It can be seen that the removal efficiency for CR, MB, or Pb(II) slightly declined with the cycles. Despite that, the adsorbents after four cycles still have a high adsorption removal efficiency for CR (79.42%), MB (75.79%), or Pb(II) (71.45%). Moreover, the SEM and XRD analyses further reveal that the architecture and phase of adsorbents after four cycles are almost retained compared with the raw adsorbent (Figures S4 and S5). These results demonstrate that the HC–MgAlLDH nanocomposite has excellent reusability.

#### 3.2.6. Removal Performance in Binary and Ternary Systems

**Figure 6:**
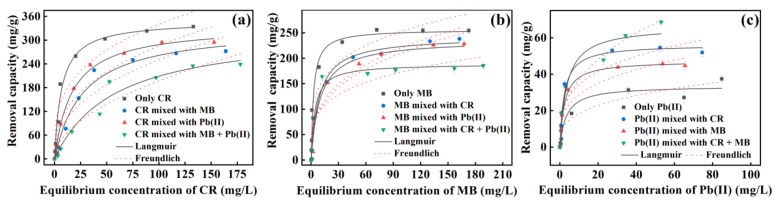
Adsorption isotherms of CR (**a**), MB (**b**) and Pb(II) (**c**) by the HC–MgAlLDH nanocomposite in the binary and ternary systems at pH 5.0, contact time 12 h, initial concentrations 10–300 mg/L for CR or MB and 1–100 mg/L for Pb(II).

Considering that CR, MB, and/or Pb(II) usually coexist in industrial wastewaters (e.g., [6,7]), the removal experiments of the binary and ternary systems were performed by varying the target concentrations with the concomitants fixed at 20 mg/L. The results are shown in Figure 6a–c, accompanied by the results for single systems for comparison. For CR at each concentration (Figure 6a), its removal capacities in the systems of CR+Pb(II), CR+MB and CR+MB+Pb(II) slightly decreased, indicating that the concomitant MB and Pb(II) have a minor effect on CR removal. The removal processes also followed the Langmuir adsorption model, with the *q*_m_ up to 337.55, 332.85 and 328.83 mg/g, respectively (Figure 6a and Table S2). Similarly, slight decreases in the *q_m_* of MB were also observed in MB+CR (242.69 mg/g), MB+Pb(II) (236.40 mg/g) and MB+CR+Pb(II) (188.96 mg/g) (Figure 6b and Table S2). Despite the declines in CR and MB removals, the HC–MgAlLDH still significantly outperforms the previous LDH-based composite adsorbents, as listed in Table S3. On the contrary, the Pb(II) removal was significantly enhanced by the CR and MB (Figure 6c), and the *q_m_* increased dramatically from 33.55 mg/g (sole Pb(II)) to 47.35 (Pb(II)+MB), 56.21 (Pb(II)+CR) and 63.28 mg/g (Pb(II)+CR+MB) (Table S2). The increases should result from the complexation of Pb(II) with the adsorbed organic dye [14] or extra precipitation of PbSO_4_ on the adsorbent. The details will be identified and discussed below. In conclusion, the results demonstrate that the HC–MgAlLDH nanocomposite could be an effective adsorbent for the simultaneous removal of CR, MB and Pb(II).

### 3.3. Insight into the Removal Mechanisms of CR, MB and Pb(II)

**Figure 7:**
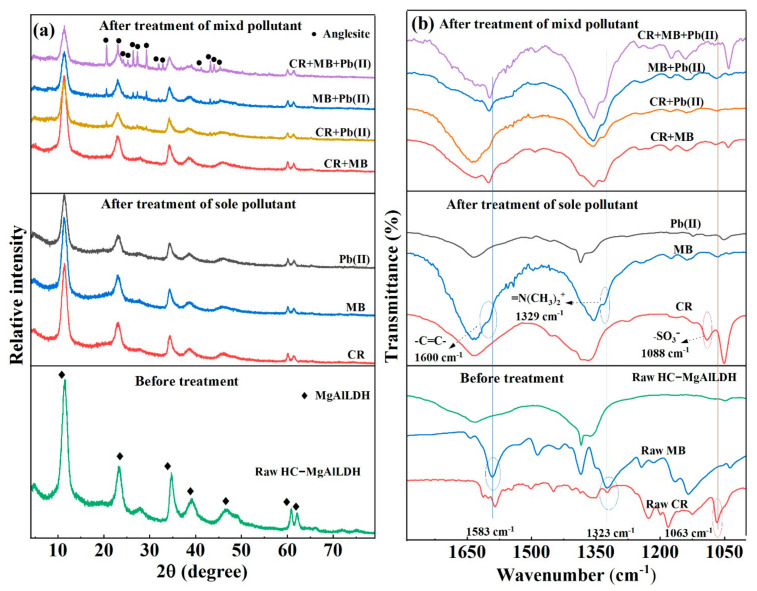
XRD patterns (**a**) and FT-IR spectra (**b**) of the HC–MgAlLDH nanocomposite before and after treating CR, MB and Pb(II).

The adsorbent HC–MgAlLDH after adsorption treatment was further characterized by XRD, FTIR, and XPS to reveal the plausible removal mechanism. After the treatment of single CR, MB and Pb(II), no discernible variations in the XRD patterns of the post-adsorbents were detected (Figure 7a), indicating that the single CR, MB and Pb(II) are removed mainly through adsorption on the nanocomposite rather than precipitation with the cations (Mg^2+^, Al^3+^) or anions (OH^−^, CO_3_^2−^) by dissolving the LDH [14,53]. In addition, the *d*_003_ values of the post-adsorbent almost remain unchanged, confirming that the dye molecules and Pb(II) ions were not intercalated into the LDH interlayer space [54]. Nevertheless, the FT-IR spectrum after CR removal (Figure 7b) shows that the S–O vibration of the sulfonic group in adsorbed CR molecules shifted from 1063 to 1088 cm^−1^, indicating that the interactions of electrostatic attraction/chemical bonding between CR and LDH component in the nanocomposite occur [16,33]. After treating MB, the band of =N(CH_3_)_2_^+^ groups in the adsorbed MB shifted from 1323 to 1329 cm^−1^ (Figure 7b), indicating that the electrostatic attraction between MB and HC component in the nanocomposite dominated the adsorption removal [55,56,57]. Additionally, the vibration of MB aromatic rings shifted from 1583 to 1600 cm^−1^ (Figure 7b), revealing that MB adsorption also occurs via the π-π interaction between the MB aromatic rings and the C=C bonds of HC component in the nanocomposite [34,55].

**Figure 8:**
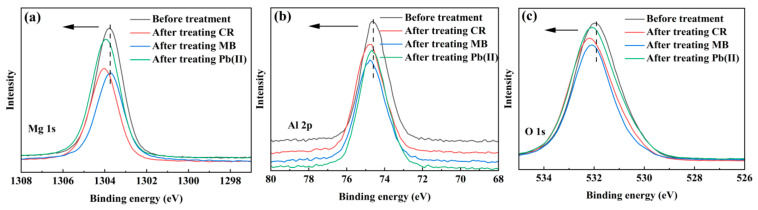
The high-resolution Mg 1s (**a**), Al 2p (**b**) and O 1s (**c**) scans of the HC–MgAlLDH nanocomposite before and after treating CR, MB and Pb(II).

In addition, the high-resolution Mg 1s, Al 2p and O 1s XPS spectra show that their binding energies all increased after CR removal (Figure 8a–c), confirming that CR was also chemically adsorbed onto the nanocomposite via the complexation of CR sulfonic groups (–SO_3_^−^) with the metal cations (i.e., Mg^2+^, Al^3+^). A similar shift in the Mg 2p binding energy of Mg(OH)_2_ caused by the complexation of Mg^2+^ with citrate –COO^−^ has also been reported [58]. After MB removal, however, only the O 1s binding energy exhibited a significant shift (Figure 8c), revealing that positively charged amino groups in MB interacted with the oxygen-containing functional groups (e.g., –COOH and –OH) of the nanocomponent HC [59]. After Pb(II) removal, obvious shifts to a high binding energy side can also be found, especially in the Mg 1s and O 1s spectra (Figure 8a–c). Similar shifts have been also observed in U(VI) adsorption on Mn_3_O_4_@sepiolite nanocomposite through Mn/Si/Mg–O–U(VI) bonds [60], and Pb(II) or Cd(II) adsorption on *Axonopus compressus*-derived biochar via their complexations with the –COOH and –OH moieties [61]. In our case, therefore, the complexations of Pb(II) with the Mg/Al–O and –COOH/–OH should be formed on the HC–MgAlLDH nanocomposite.

Unlike the results for the removal of the sole pollutant, all the of XRD patterns of the post-adsorbents after treatment of the Pb(II)-containing binary and ternary systems show a set of new peaks that can be well indexed to anglesite (PbSO_4_, JCPDS file 36-1461) (Figure 7a). This confirms that the formation of PbSO_4_ precipitation is an additional removal mechanism for Pb(II) in the binary and ternary solutions. The SO_4_^2−^ should originate from the desulfonation of CR and/or the oxidation of the thiazine ring in MB caused by the HC-generated free radicals [62]. Moreover, the diffraction intensity of PbSO_4_ was more intense after treatment of the ternary system than the binary systems (Figure 7a), indicating that more PbSO_4_ was formed in the presence of the two dyes. This should be ascribed to more SO_4_^2−^ supply by the coexisting dyes. The PbSO_4_ precipitation can further increase the removal capacity of Pb(II), supporting the high removal results (Figure 6c). It appears that the HC–MgAlLDH nanocomposite possesses much more specificity for multiple pollutant removal. Besides, the FT-IR spectra after the treatment of binary and ternary pollutants show similar shifts to those after the sole treatment (Figure 7b), indicating that the similar electrostatic attraction/chemical bonding contributes significantly to the multi-pollutant mixture removal as well.

**Figure 9:**
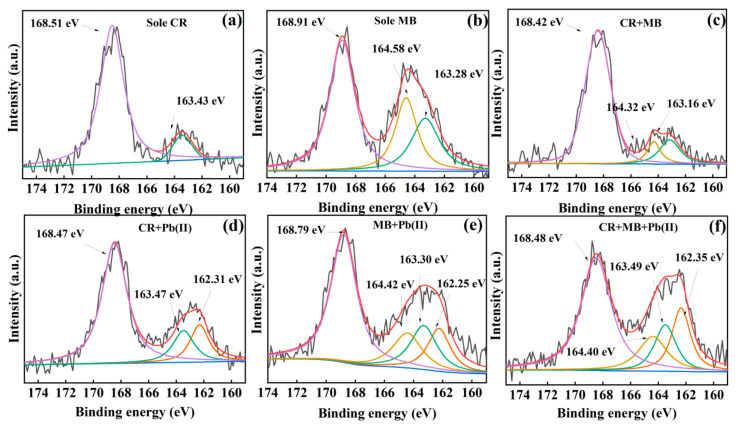
High-resolution S 2p XPS spectra of the HC–MgAlLDH after treating various systems: CR (**a**), MB (**b**), CR+MB (**c**), CR+Pb(II) (**d**), MB+Pb(II) (**e**) and CR+MB+Pb(II) (**f**).

To further explore the interactions of the S-bearing groups in CR and MB with the nanocomposite and Pb(II) during the removal, analyses on the high-resolution S 2p XPS spectra were conducted after the adsorptions (Figure 9). For the CR-adsorbed sample (Figure 9a), the S 2p spectrum shows a main peak at 168.51 eV assigned to the –SO_3_^−^ of CR and a weak peak corresponding to thiol-type S at a lower energy of 163.43 eV [63,64], indicating that a small amount of CR on the adsorbent was reduced to thiols by the nanocomposite. As to the MB-adsorbed sample (Figure 9b), in addition to the peak of the C–S bond in MB at 164.58 eV, the peaks of oxidized-state S–O bond at a higher energy (168.91 eV) and reduced-state S–H bond at a lower energy (163.28 eV) can be clearly distinguished, indicating that the C–S bonds in the adsorbed MB were disproportionated. Previous studies have confirmed that HC with rich organic groups can act as the electron shuttle to induce the abiotic reduction of heavy metals ions (e.g., Cr(VI) and Ag(I)) and generate reactive oxygen species (e.g., •O_2_^−^, H_2_O_2_, and •OH) to oxidize organic pollutant (e.g., sulfadimidine and bisphenols) [65,66,67]. Therefore, in our case, the CR reduction and MB disproportionation should be associated with the HC in the nanocomposite. The S 2p spectrum after the removal of coexisting CR and MB (Figure 9c) shows an integrated state of the spectra after their single removal. Nevertheless, after treating the Pb(II)-containing binary and ternary solutions, the S 2p binding energies associated with thiol (163.28–163.43 eV) shifted to lower energy ranges (162.25–162.35 eV) (Figure 9d–f), indicating that Pb(II)–thiol complex was formed on the adsorbent [68]. In conclusion, the removal of CR, MB and Pb(II) by the HC–MgAlLDH nanocomposite involves multiple actions, including electrostatic adsorption, π–π interaction, coordination and/or precipitation crystallization.

## 4. Conclusions

In summary, the HC–MgAlLDH nanocomposite was successfully fabricated by a facile one-step hydrothermal technique and used as an adsorbent for single or simultaneous removal of anionic dye CR and cationic dye MB as well as heavy metal Pb(II). The batch removal experimental results revealed that the nanocomposite has superior removal performances to the single MgAlLDH and HC, and its maximum Langmuir removal capacity to CR, MB or Pb(II) is 348.78, 256.54, or 33.55 mg/g, respectively. In the multi-pollutant systems, the nanocomposite can not only dramatically increase Pb(II) removal, but also exhibits high removal of CR and MB. Therefore, the nanocomposite can act as an effective adsorbent for the simultaneous removal of CR, MB and Pb(II). Moreover, the removal of CR, MB and Pb(II) by the nanocomposite involves a series of interactions between the adsorbate species and the adsorbent, including electrostatic adsorption, π–π interaction, coordination and/or precipitation crystallization. Current results contribute to the efficient removal of various types of pollutants by developing a new and multifunctional adsorbent and advance the understanding of the interactions between heavy metals and organic dyes during wastewater treatment.

## Data Availability

The data presented in this study are available on request from the corresponding authors.

## Supplementary Materials

The following supporting information can be downloaded at: https://www.mdpi.com/article/10.3390/nano13071145/s1, Text S1: Adsorption kinetic and isothermal models; Table S1: Kinetic parameters of CR, MB or Pb(II) adsorption onto the HC–MgAlLDH; Table S2: Adsorption isothermal parameters of CR, MB and Pb(II) by the HC–MgAlLDH nanocomposite in various systems; Table S3: Comparison of the Langmuir removal capacities (*q*_m_) of various LDH-based adsorbents to single CR, MB and Pb(II); Figure S1: SEM images and EDS analyses of the synthesized MgAlLDH (a) and HC (b); Figure S2: The species distribution of Pb(II) in aqueous solution (50 mg/L Pb(II)), calculated by a chemical equilibrium program Visual MINTEQ (version 3.1); Figure S3: Recycle test for the removal of CR, MB and Pb(II) by HC–MgAlLDH nanocomposite; Figure S4: SEM images of regenerated HC–MgAlLDH nanocomposite after treatment of CR (a), MB (b) and Pb(II) (c); Figure S5: XRD patterns of regenerated HC–MgAlLDH nanocomposite after treatment of CR (a), MB (b) or Pb(II) (c).

## References

[1] Afkhami A., Moosavi R. (2010). Adsorptive Removal of Congo Red, a Carcinogenic Textile Dye, from Aqueous Solutions by Maghemite Nanoparticles. J. Hazard. Mater..

[2] Jha P., Jobby R., Desai N.S. (2016). Remediation of Textile Azo Dye Acid Red 114 by Hairy Roots of Ipomoea Carnea Jacq. and Assessment of Degraded Dye Toxicity with Human Keratinocyte Cell Line. J. Hazard. Mater..

[3] Cheng R., Ou S., Xiang B., Li Y., Liao Q. (2010). Equilibrium and Molecular Mechanism of Anionic Dyes Adsorption onto Copper(II) Complex of Dithiocarbamate-Modified Starch. Langmuir.

[4] Yaseen D.A., Scholz M. (2019). Textile Dye Wastewater Characteristics and Constituents of Synthetic Effluents: A Critical Review. Int. J. Environ. Sci. Technol..

[5] Yaseen D.A., Scholz M. (2018). Treatment of Synthetic Textile Wastewater Containing Dye Mixtures with Microcosms. Environ. Sci. Pollut. Res..

[6] Yang L., Zhang Y., Liu X., Jiang X., Zhang Z., Zhang T., Zhang L. (2014). The Investigation of Synergistic and Competitive Interaction between Dye Congo Red and Methyl Blue on Magnetic MnFe_2_O_4_. Chem. Eng. J..

[7] İşmal Ö.E., Yıldırım L. (2019). Metal Mordants and Biomordants. The Impact and Prospects of Green Chemistry for Textile Technology.

[8] Holkar C.R., Jadhav A.J., Pinjari D.V., Mahamuni N.M., Pandit A.B. (2016). A Critical Review on Textile Wastewater Treatments: Possible Approaches. J. Environ. Manag..

[9] Jadhav J.P., Kalyani D.C., Telke A.A., Phugare S.S., Govindwar S.P. (2010). Evaluation of the Efficacy of a Bacterial Consortium for the Removal of Color, Reduction of Heavy Metals, and Toxicity from Textile Dye Effluent. Bioresour. Technol..

[10] Ali H., Khan E., Ilahi I. (2019). Environmental Chemistry and Ecotoxicology of Hazardous Heavy Metals: Environmental Persistence, Toxicity, and Bioaccumulation. J. Chem..

[11] Shanker U., Rani M., Jassal V. (2017). Degradation of Hazardous Organic Dyes in Water by Nanomaterials. Environ. Chem. Lett..

[12] Ling C., Liu F.-Q., Long C., Chen T.-P., Wu Q.-Y., Li A.-M. (2014). Synergic Removal and Sequential Recovery of Acid Black 1 and Copper (II) with Hyper-Crosslinked Resin and inside Mechanisms. Chem. Eng. J..

[13] Sharma S., Sharma S., Upreti N., Sharma K.P. (2009). Monitoring Toxicity of an Azo Dye Methyl Red and a Heavy Metal Cu, Using Plant and Animal Bioassays. Toxicol. Environ. Chem..

[14] Chen Y.-Y., Yu S.-H., Jiang H.-F., Yao Q.-Z., Fu S.-Q., Zhou G.-T. (2018). Performance and Mechanism of Simultaneous Removal of Cd(II) and Congo Red from Aqueous Solution by Hierarchical Vaterite Spherulites. Appl. Surf. Sci..

[15] Bharali D., Deka R.C. (2017). Preferential Adsorption of Various Anionic and Cationic Dyes from Aqueous Solution over Ternary CuMgAl Layered Double Hydroxide. Colloids Surf. A Physicochem. Eng..

[16] Ahmed I.M., Gasser M.S. (2012). Adsorption Study of Anionic Reactive Dye from Aqueous Solution to Mg–Fe–CO_3_ Layered Double Hydroxide (LDH). Appl. Surf. Sci..

[17] Moraes P.I.R., Tavares S.R., Vaiss V.S., Leitão A.A. (2018). Investigation on Sustainable Phosphate Release in Agriculture: Structural and Thermodynamic Study of Stability, Dehydration and Anionic Exchange of Mg-Al-HPO_4_ Layered Double Hydroxide by DFT Calculations. Appl. Clay Sci..

[18] Yang Z., Wang F., Zhang C., Zeng G., Tan X., Yu Z., Zhong Y., Wang H., Cui F. (2016). Utilization of LDH-Based Materials as Potential Adsorbents and Photocatalysts for the Decontamination of Dyes Wastewater: A Review. RSC Adv..

[19] Nguyen T.H., Tran H.N., Nguyen T.V., Vigneswaran S., Trinh V.T., Nguyen T.D., Ha Nguyen T.H., Mai T.N., Chao H.-P. (2022). Single-Step Removal of Arsenite Ions from Water through Oxidation-Coupled Adsorption Using Mn/Mg/Fe Layered Double Hydroxide as Catalyst and Adsorbent. Chemosphere.

[20] Jiang D.B., Jing C., Yuan Y., Feng L., Liu X., Dong F., Dong B., Zhang Y.X. (2019). 2D-2D Growth of NiFe LDH Nanoflakes on Montmorillonite for Cationic and Anionic Dye Adsorption Performance. J. Colloid Interface Sci..

[21] Kambo H.S., Dutta A. (2015). A Comparative Review of Biochar and Hydrochar in Terms of Production, Physico-Chemical Properties and Applications. Renew. Sustain. Energy Rev..

[22] Zhang Z., Zhu Z., Shen B., Liu L. (2019). Insights into Biochar and Hydrochar Production and Applications: A Review. Energy.

[23] Rathinavel S., Priyadharshini K., Panda D. (2021). A Review on Carbon Nanotube: An Overview of Synthesis, Properties, Functionalization, Characterization, and the Application. Mater. Sci. Eng. B.

[24] Malool M.E., Keshavarz Moraveji M., Shayegan J. (2021). Optimized Production, Pb(II) Adsorption and Characterization of Alkali Modified Hydrochar from Sugarcane Bagasse. Sci. Rep..

[25] Nadarajah K., Bandala E.R., Zhang Z., Mundree S., Goonetilleke A. (2021). Removal of Heavy Metals from Water Using Engineered Hydrochar: Kinetics and Mechanistic Approach. J. Water Process Eng..

[26] Xia Y., Yang T., Zhu N., Li D., Chen Z., Lang Q., Liu Z., Jiao W. (2019). Enhanced Adsorption of Pb(II) onto Modified Hydrochar: Modeling and Mechanism Analysis. Bioresour. Technol..

[27] Li B., Lv J.-Q., Guo J.-Z., Fu S.-Y., Guo M., Yang P. (2019). The Polyaminocarboxylated Modified Hydrochar for Efficient Capturing Methylene Blue and Cu(II) from Water. Bioresour. Technol..

[28] Li H.-Z., Zhang Y.-N., Guo J.-Z., Lv J.-Q., Huan W.-W., Li B. (2021). Preparation of Hydrochar with High Adsorption Performance for Methylene Blue by Co-Hydrothermal Carbonization of Polyvinyl Chloride and Bamboo. Bioresour. Technol..

[29] Memon N., Kanwal U., Memon A., Memon S.S., Memon S.Q. (2021). Synthesis, Characterization, and Application of Co-Al-Zn Layered Double Hydroxide/Hydrochar Composite for Simultaneous Removal of Cationic and Anionic Dyes. J. Chem..

[30] Dat N.D., Loc T.T., Trieu M.T., Nguyen D.T., Nguyen K.Q., Nguyen M.L., Le A.D.D., Tran H.N. (2022). Composites Derived from Synthetic Clay and Carbon Sphere: Preparation, Characterization, and Application for Dye Decontamination. Korean J. Chem. Eng..

[31] Zhang J.-W., Nur’aini A.D., Wang Y.-C., Hai N.D., Van Minh D., Chao H.-P. (2022). Multiple Pollutants Removal by Carbon Sphere and Layered Double Hydroxide Composites: Adsorption Behavior and Mechanisms. J. Environ. Chem. Eng..

[32] Ischia G., Cutillo M., Guella G., Bazzanella N., Cazzanelli M., Orlandi M., Miotello A., Fiori L. (2022). Hydrothermal Carbonization of Glucose: Secondary Char Properties, Reaction Pathways, and Kinetics. Chem. Eng. J..

[33] Lafi R., Charradi K., Djebbi M.A., Ben Haj Amara A., Hafiane A. (2016). Adsorption Study of Congo Red Dye from Aqueous Solution to Mg–Al–Layered Double Hydroxide. Adv. Powder Technol..

[34] Xiong T., Yuan X., Chen X., Wu Z., Wang H., Leng L., Wang H., Jiang L., Zeng G. (2018). Insight into Highly Efficient Removal of Cadmium and Methylene Blue by Eco-Friendly Magnesium Silicate-Hydrothermal Carbon Composite. Appl. Surf. Sci..

[35] Chen S., Huang Y., Han X., Wu Z., Lai C., Wang J., Deng Q., Zeng Z., Deng S. (2018). Simultaneous and Efficient Removal of Cr(VI) and Methyl Orange on LDHs Decorated Porous Carbons. Chem. Eng. J..

[36] Latham K.G., Jambu G., Joseph S.D., Donne S.W. (2014). Nitrogen Doping of Hydrochars Produced Hydrothermal Treatment of Sucrose in H_2_O, H_2_SO_4_, and NaOH. ACS Sustain. Chem. Eng..

[37] Zhang R., Chen C., Li J., Wang X. (2015). Preparation of Montmorillonite@carbon Composite and Its Application for U(VI) Removal from Aqueous Solution. Appl. Surf. Sci..

[38] Jiménez-López B.A., Leyva-Ramos R., Salazar-Rábago J.J., Jacobo-Azuara A., Aragón-Piña A. (2021). Adsorption of Selenium (IV) Oxoanions on Calcined Layered Double Hydroxides of Mg-Al-CO_3_ from Aqueous Solution. Effect of Calcination and Reconstruction of Lamellar Structure. Environ. Nanotechnol. Monit. Manag..

[39] Yin W., Liu M., Zhao T.-L., Qian F.-J., Li H., Yao Q.-Z., Fu S.-Q., Zhou G.-T. (2020). Removal and Recovery of Silver Nanoparticles by Hierarchical Mesoporous Calcite: Performance, Mechanism, and Sustainable Application. Environ. Res..

[40] Feng L., Zhang Q., Ji F., Jiang L., Liu C., Shen Q., Liu Q. (2022). Phosphate Removal Performances of Layered Double Hydroxides (LDH) Embedded Polyvinyl Alcohol/Lanthanum Alginate Hydrogels. Chem. Eng. J..

[41] Tran H.N., You S.-J., Hosseini-Bandegharaei A., Chao H.-P. (2017). Mistakes and Inconsistencies Regarding Adsorption of Contaminants from Aqueous Solutions: A Critical Review. Water Res..

[42] Liu M., Yin W., Qian F.-J., Zhao T.-L., Yao Q.-Z., Fu S.-Q., Zhou G.-T. (2020). A Novel Synthesis of Porous TiO_2_ Nanotubes and Sequential Application to Dye Contaminant Removal and Cr(VI) Visible Light Catalytic Reduction. J. Environ. Chem. Eng..

[43] Jia Y.-H., Liu Z.-H. (2019). Preparation of Borate Anions Intercalated MgAl-LDHs Microsphere and Its Calcinated Product with Superior Adsorption Performance for Congo Red. Colloids Surf. A Physicochem. Eng..

[44] Sun Q., Tang M., Hendriksen P.V., Chen B. (2020). Biotemplated Fabrication of a 3D Hierarchical Structure of Magnetic ZnFe_2_O_4_/MgAl-LDH for Efficient Elimination of Dye from Water. J. Alloys Compd..

[45] Sriram G., Uthappa U.T., Losic D., Kigga M., Jung H.-Y., Kurkuri M.D. (2020). Mg–Al-Layered Double Hydroxide (LDH) Modified Diatoms for Highly Efficient Removal of Congo Red from Aqueous Solution. Appl. Sci..

[46] Nazir M.A., Khan N.A., Cheng C., Shah S.S.A., Najam T., Arshad M., Sharif A., Akhtar S., Rehman A. (2020). Surface Induced Growth of ZIF-67 at Co-Layered Double Hydroxide: Removal of Methylene Blue and Methyl Orange from Water. Appl. Clay Sci..

[47] Starukh G., Rozovik O., Oranska O. (2016). Organo/Zn-Al LDH Nanocomposites for Cationic Dye Removal from Aqueous Media. Nanoscale Res. Lett..

[48] Bo L., Li Q., Wang Y., Gao L., Hu X., Yang J. (2015). One-Pot Hydrothermal Synthesis of Thrust Spherical Mg–Al Layered Double Hydroxides/MnO_2_ and Adsorption for Pb(II) from Aqueous Solutions. J. Environ. Chem. Eng..

[49] Yasin Y., Mohamad M., Saad A., Sanusi A., Ahmad F.H. (2014). Removal of Lead Ions from Aqueous Solutions Using Intercalated Tartrate-Mg–Al Layered Double Hydroxides. Desalination Water Treat..

[50] Hu H., Wageh S., Al-Ghamdi A.A., Yang S., Tian Z., Cheng B., Ho W. (2020). NiFe-LDH Nanosheet/Carbon Fiber Nanocomposite with Enhanced Anionic Dye Adsorption Performance. Appl. Surf. Sci..

[51] Missau J., Bertuol D.A., Tanabe E.H. (2021). Highly Efficient Adsorbent for Removal of Crystal Violet Dye from Aqueous Solution by CaAl/LDH Supported on Biochar. Appl. Clay Sci..

[52] Hou T., Yan L., Li J., Yang Y., Shan L., Meng X., Li X., Zhao Y. (2020). Adsorption Performance and Mechanistic Study of Heavy Metals by Facile Synthesized Magnetic Layered Double Oxide/Carbon Composite from Spent Adsorbent. Chem. Eng. J..

[53] Liu M., Yin W., Zhao T.-L., Yao Q.-Z., Fu S.-Q., Zhou G.-T. (2021). High-Efficient Removal of Organic Dyes from Model Wastewater Using Mg(OH)_2_-MnO_2_ Nanocomposite: Synergistic Effects of Adsorption, Precipitation, and Photodegradation. Sep. Purif. Technol..

[54] Zhang H., Zhou J., Muhammad Y., Tang R., Liu K., Zhu Y., Tong Z. (2019). Citric Acid Modified Bentonite for Congo Red Adsorption. Front. Mater..

[55] Li B., Guo J., Lv K., Fan J. (2019). Adsorption of Methylene Blue and Cd(II) onto Maleylated Modified Hydrochar from Water. Environ. Pollut..

[56] Wu Z., Zhong H., Yuan X., Wang H., Wang L., Chen X., Zeng G., Wu Y. (2014). Adsorptive Removal of Methylene Blue by Rhamnolipid-Functionalized Graphene Oxide from Wastewater. Water Res..

[57] Zhuo Q., Ma H., Wang B., Fan F. (2008). Degradation of Methylene Blue: Optimization of Operating Condition through a Statistical Technique and Environmental Estimate of the Treated Wastewater. J. Hazard. Mater..

[58] Yin W., Liu M., Wang Y.-H., Huang Y., Zhao T.-L., Yao Q.-Z., Fu S.-Q., Zhou G.-T. (2022). Fe_3_O_4_–Mg(OH)_2_ Nanocomposite as a Scavenger for Silver Nanoparticles: Rational Design, Facile Synthesis, and Enhanced Performance. Environ. Res..

[59] Yang Z., Hou J., Miao L., Wu J. (2021). Comparison of Adsorption Behavior Studies of Methylene Blue by Microalga Residue and Its Biochars Produced at Different Pyrolytic Temperatures. Environ. Sci. Pollut. Res..

[60] Yin W., Liu M., Chen Y.-Y., Yao Q.-Z., Fu S.-Q., Zhou G.-T. (2022). Microwave-Assisted Preparation of Mn_3_O_4_@sepiolite Nanocomposite for Highly Efficient Removal of Uranium. Appl. Clay Sci..

[61] Yu W., Hu J., Yu Y., Ma D., Gong W., Qiu H., Hu Z., Gao H. (2021). Facile Preparation of Sulfonated Biochar for Highly Efficient Removal of Toxic Pb(II) and Cd(II) from Wastewater. Sci. Total Environ..

[62] Chen N., Huang Y., Hou X., Ai Z., Zhang L. (2017). Photochemistry of Hydrochar: Reactive Oxygen Species Generation and Sulfadimidine Degradation. Environ. Sci. Technol..

[63] Castner D.G., Hinds K., Grainger D.W. (1996). X-Ray Photoelectron Spectroscopy Sulfur 2p Study of Organic Thiol and Disulfide Binding Interactions with Gold Surfaces. Langmuir.

[64] Klem E.J.D., MacNeil D.D., Levina L., Sargent E.H. (2008). Solution Processed Photovoltaic Devices with 2% Infrared Monochromatic Power Conversion Efficiency: Performance Optimization and Oxide Formation. Adv. Mater..

[65] Chen N., Cao S., Zhang L., Peng X., Wang X., Ai Z., Zhang L. (2021). Structural Dependent Cr(VI) Adsorption and Reduction of Biochar: Hydrochar versus Pyrochar. Sci. Total Environ..

[66] Ruan X., Sun Y., Du W., Tang Y., Liu Q., Zhang Z., Doherty W., Frost R.L., Qian G., Tsang D.C.W. (2019). Formation, Characteristics, and Applications of Environmentally Persistent Free Radicals in Biochars: A Review. Bioresour. Technol..

[67] Yan Y., Ma X., Cao W., Zhang X., Zhou J., Liu Q., Qian G. (2018). Identifying the Reducing Capacity of Biomass Derived Hydrochar with Different Post-Treatment Methods. Sci. Total Environ..

[68] Liang X., Xu Y., Sun G., Wang L., Sun Y., Qin X. (2009). Preparation, Characterization of Thiol-Functionalized Silica and Application for Sorption of Pb^2+^ and Cd^2+^. Colloids Surf. A Physicochem. Eng..

