# Supplementary material for: Efficient Removal of Congo Red, Methylene Blue and Pb(II) by Hydrochar–MgAlLDH Nanocomposite: Synthesis, Performance and Mechanism
Source: Nanomaterials (Basel). 2023 Mar 23;13(7):1145. doi: 10.3390/nano13071145 (PMC10096714; doi:10.3390/nano13071145)
Supplement: Supplementary file 1 [file nanomaterials-13-01145-s001.zip › nanomaterials-2226670-supplementary.pdf]

nanomaterials

MDPI

Supplementary Materials

Efficient Removal of Congo Red, Methylene Blue and Pb(II) by Hydrochar–MgAlLDH Nanocomposite: Synthesis, Performance and Mechanism

Yang Huang [1,2], Wei Yin [1], Tian-Lei Zhao [1], Meng Liu [1], Qi-Zhi Yao [3] and Gen-Tao Zhou [1,4,*]

1 CAS Key Laboratory of Crust-Mantle Materials and Environments, School of Earth and Space Sciences, University of Science and Technology of China, Hefei 230026, China; esthy@mail.ustc.edu.cn (Y.H.)
2 School of Environmental Engineering and Resources, University of Science and Technology of Southwest, Mianyang 621010, China
3 School of Chemistry and Materials Science, University of Science and Technology of China, Hefei 230026, China
4 CAS Center for Excellence in Comparative Planetology, University of Science and Technology of China, Hefei 230026, China
* Correspondence: gtzhou@ustc.edu.cn; Tel.: +86-551-63600533

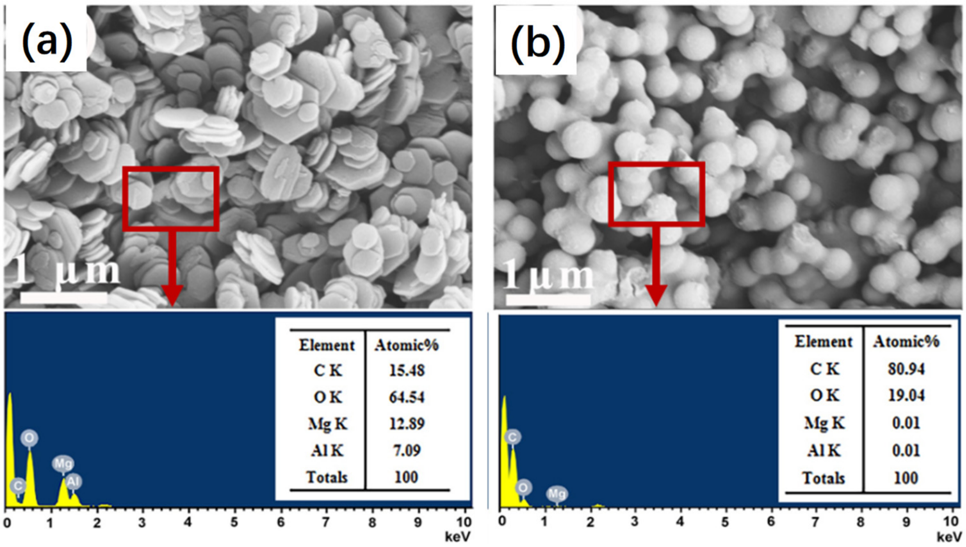

Figure S1. SEM images and EDS analyses of the synthesized MgAlLDH (a) and HC (b).

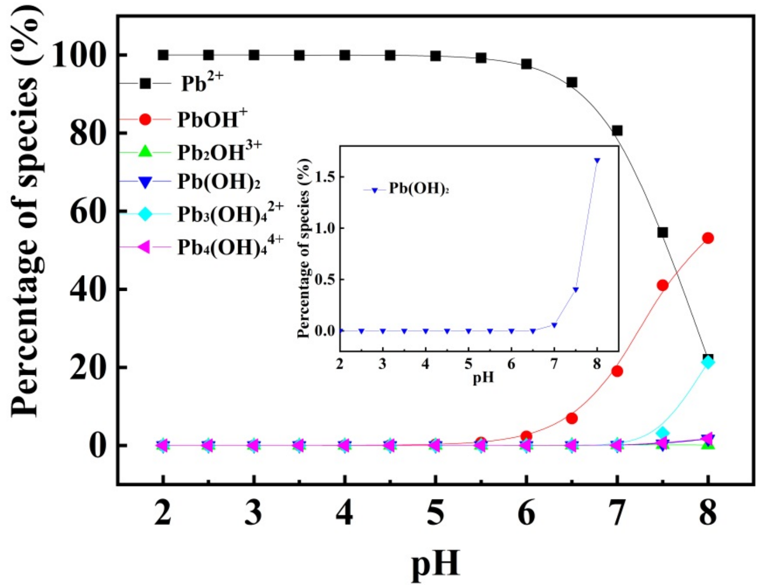

Figure S2. The species distribution of Pb(II) in aqueous solution (50 mg/L Pb(II)), calculated by a chemical equilibrium program Visual MINTEQ (version 3.1).

TEXT S1 Adsorption kinetic and isothermal models

Adsorption data at different contact times were fitted with pseudo-first-order (Equation S1) and pseudo-second-order (Equation S2) kinetics to determine the kinetic parameters [38]:

$$q_t = q_e(1 - \exp(-k_1 t)) \quad \text{(S1)}$$

$$q_t = \frac{k_2 q_e^2 t}{1 + k_2 q_e t} \quad \text{(S2)}$$

where q_e and q_t are the amounts of pollutants adsorbed (mg/g) at equilibrium and at time t (min), k_1 is the pseudo-first-order rate constant (min^{-1}), and k_2 (g/mg/min) is the rate constant of the pseudo-second-order kinetics.

The Langmuir and Freundlich isothermal models are expressed by Equations S3 and S4, respectively [38]:

$$q_e = q_m b C_e/(1 + bC_e) \quad \text{(S3)}$$

$$q_e = K_f C_e^{1/n} \quad \text{(S4)}$$

where constant b is related to the energy of adsorption (L/mg), q_m is the maximum adsorption capacity (mg/g), K_f is an indicator of the adsorption capacity, and $1/n$ is the adsorption intensity.

Table S1. Kinetic parameters of CR, MB or Pb(II) adsorption onto the HC−MgAlLDH.

**Pollutants**	$q_{e,exp}$ **(mg/g)**	**Pseudo-First-Order**			**Pseudo-Second-Order**		
		k_1 **(min^{-1})**	$q_{e,cal}$ **(mg/g)**	R^2	k_2 **(g/(mg·min))**	$q_{e,cal}$ **(mg/g)**	R^2
CR	172.85	0.02709	156.99	0.9419	2.33×10^{-4}	167.36	0.9835
MB	161.67	0.1795	153.06	0.9670	0.0023	157.19	0.9869
Pb(II)	38.44	0.0787	35.49	0.9491	0.0034	37.05	0.9897

Table S2. Adsorption isothermal parameters of CR, MB and Pb(II) by the HC−MgAlLDH nanocomposite in various systems.

Pollutant	Mono/multi-component system	Langmuir Model			Freundlich Model		
		q_m (mg/g)	b (L/mg)	R^2	K_f(mg/g)	n	R^2
**CR**	Only CR	348.78	0.1497	0.9857	82.9671	3.2636	0.9030
	CR+MB	332.85	0.03667	0.9793	36.0747	2.3752	0.9008
	CR+Pb(II)	337.55	0.05887	0.9938	50.0129	2.6443	0.9168
	CR+MB+Pb(II)	328.83	0.01741	0.9641	18.3591	1.9401	0.9259
MB	Only MB	256.54	0.3851	0.9799	86.7985	4.3003	0.9002
	MB+CR	242.69	0.1153	0.9706	49.2889	3.0694	0.9174
	MB+Pb(II)	236.45	0.1028	0.9673	46.7023	3.0525	0.9162
	MB+CR+Pb(II)	188.96	0.2443	0.9491	55.0183	4.0120	0.8116
Pb(II)	Only Pb(II)	33.55	0.2950	0.9376	9.0174	3.2201	0.9260
	Pb(II)+CR	56.21	0.4520	0.9561	18.5252	3.7301	0.8664
	Pb(II)+MB	47.35	0.4135	0.9754	14.9293	3.5191	0.8921
	Pb(II)+CR+MB	63.28	0.2867	0.8863	14.4048	2.5713	0.9108

Table S3. Comparison of the Langmuir removal capacities (q_m) of various LDH-based adsorbents to single CR, MB and Pb(II).

Sorbate	Adsorbent	Experimental Conditions	q_m (mg/g)	Ref.
CR	MgAlLDH	T=25°C	111.11	[33]
	Borate intercalated MgAlLDH	—	166.39	[43]
	$ZnFe_2O_4$/MgAlLDH	T=25°C	294.12	[44]
	MgAlLDH modified diatom	pH=7	305.80	[45]
	HC−MgAlLDH	pH=5, T=25°C	348.78	This work
MB	ZIF-67/CoAlLDH	T=30°C	57.24	[46]
	NiFeLDH decorated montmorillonite	T=25°C	99.18	[20]
	Dodecyl sulfate modified ZnAlLDH	—	113.00	[47]
	Hydrochar capped MgAlLDH	pH=7, T=30°C	226.00	[30]
	HC−MgAlLDH	pH=5, T=25°C	256.54	This work
Pb(II)	MgAlLDH	T=25°C	16.93	[48]
	MgFeLDH	T=25°C	18.45	[19]
	Tartrate intercalated MgAlLDH	—	8.40	[49]
	HC−MgAlLDH	pH=5, T=25°C	33.55	This work

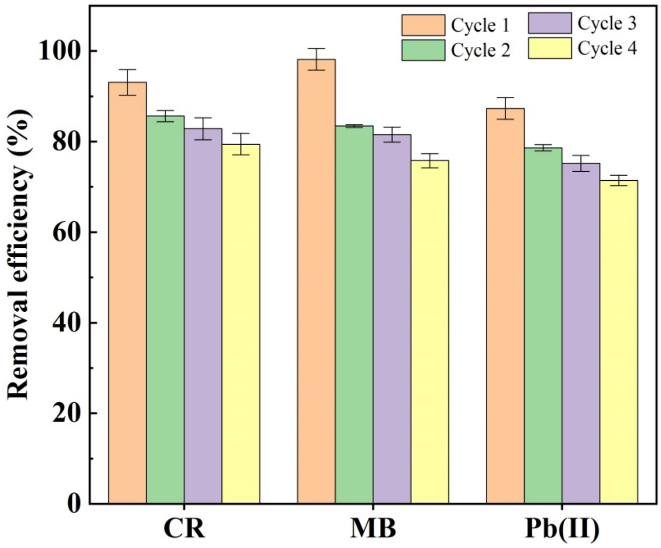

Figure S3. Recycle test for the removal of CR, MB and Pb(II) by HC−MgAlLDH nanocomposite.

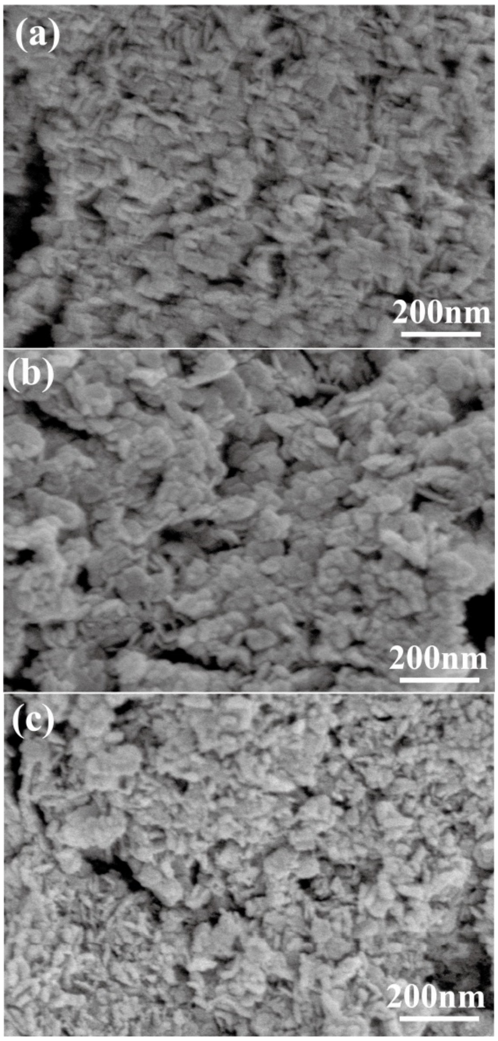

Figure S4. SEM images of regenerated HC-MgAlLDH nanocomposite after treatment of CR (**a**), MB (**b**) and Pb(II) (**c**).

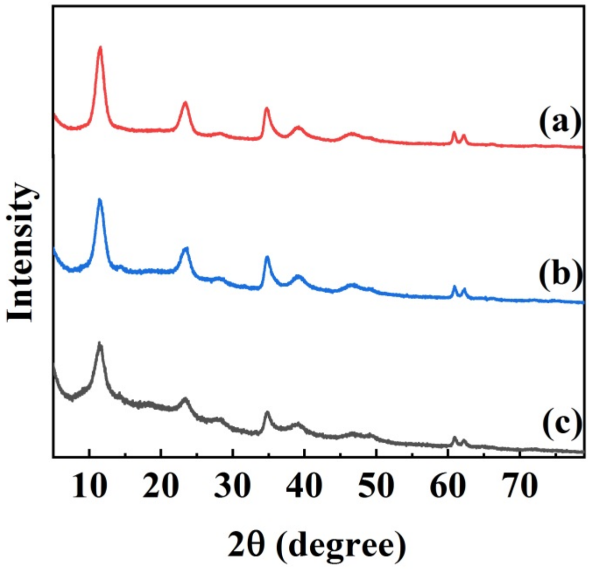

Figure S5. XRD patterns of regenerated HC-MgAlLDH nanocomposite after treatment of CR (**a**), MB (**b**) and Pb(II) (**c**).
